# Supplementary material for: Identification and characterisation of an elusive bacterial enzyme system for chloromethane dehalogenation
Source: Nat Commun. 2026 May 30;17:4818. doi: 10.1038/s41467-026-73764-z (PMC13222342; doi:10.1038/s41467-026-73764-z)
Supplement: Supplementary file 1 — Supplementary Information [file 41467_2026_73764_MOESM1_ESM.pdf]

Supplementary Information

Identification and characterisation of an elusive bacterial enzyme system for chloromethane dehalogenation

Jasmin Bernhardt[1,2,3], Lukas K. R. Hofmann[1,2,3,4], Paul Klemm[5], Nicole Paczia[4], Olivier N. Lemaire[6,7], Stéphane Vuilleumier[8], Tristan Wagner[6,7], Julia M. Kurth[1,2,3,5]

[1] Institute for Molecular Microbiology and Biotechnology, University of Münster, Münster, Germany

[2] Microcosm Earth Center, University of Marburg and Max Planck Institute for Terrestrial Microbiology, Marburg, Germany

[3] Microbial Physiology Lab, Department of Chemistry, University of Marburg, Marburg, Germany

[4] Max Planck Institute for Terrestrial Microbiology, Marburg, Germany

[5] Center for Synthetic Microbiology (SYNMIKRO), Marburg, Germany

[6] Institut de Biologie Structurale, CEA, CNRS, Université Grenoble Alpes, Grenoble, France

[7] Max Planck Institute for Marine Microbiology, Bremen, Germany

[8] Génétique Moléculaire, Génomique, Microbiologie, UMR 7156 CNRS, Université de Strasbourg, Strasbourg, France

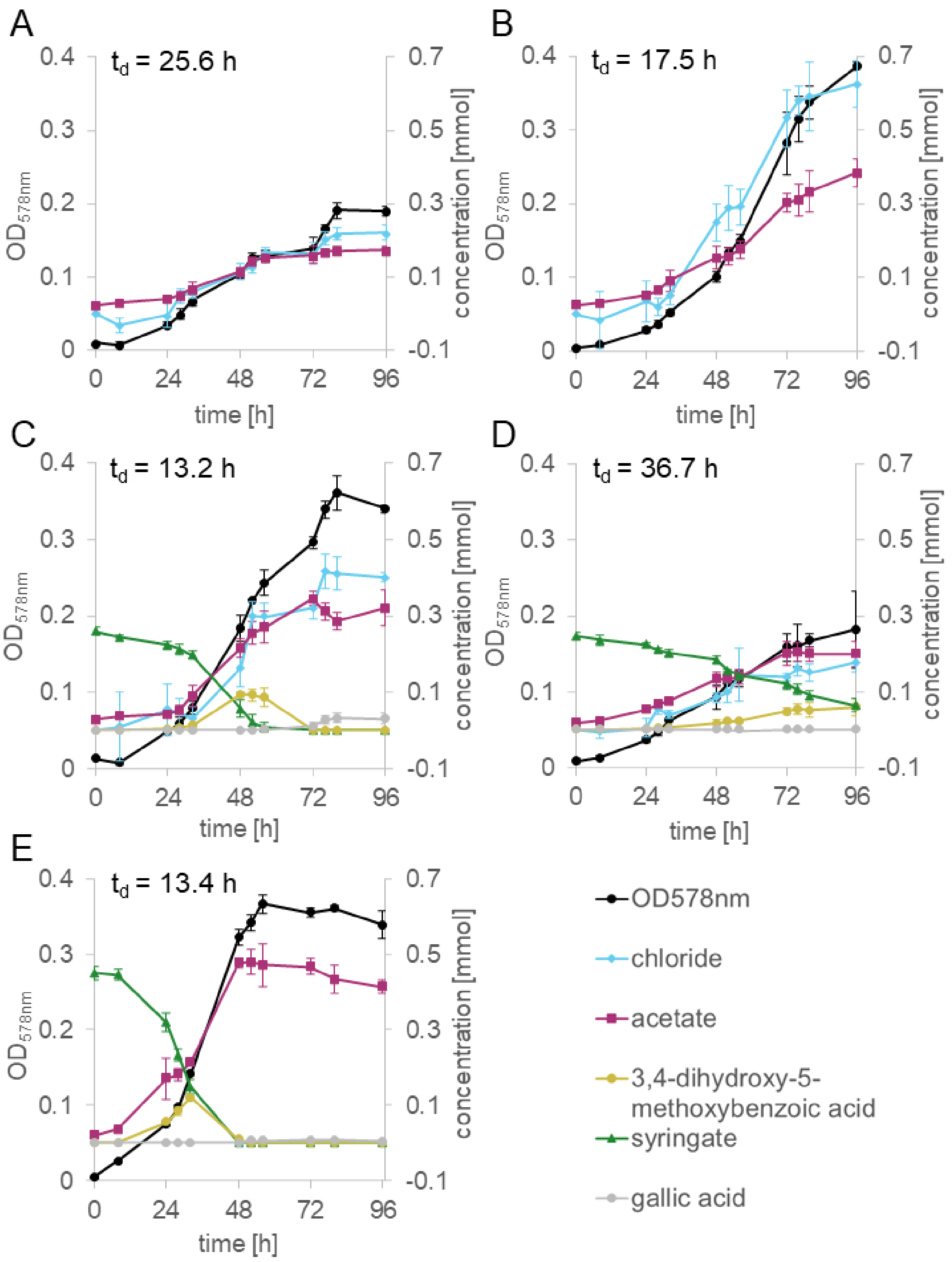

Supplementary Figure 1. Growth of *A. dehalogenans* on syringate (Syr), chloromethane (CM), and both substrates. Growth curves show *A. dehalogenans* grown with 0.65 mmol CM (**A**), 1.72 mmol CM (**B**), 0.25 mmol syringate (5 mM) and 0.65 mmol CM (**C**), 0.25 mmol (5 mM) syringate and 1.72 mmol CM (**D**), and 0.5 mmol (10 mM) syringate (**E**) in a 120 ml bottle with 50 ml medium. The following parameters were measured: OD_{578nm} (black circles), acetate (purple squares), chloride (blue diamonds), syringate (green triangles), 3,4-dihydroxy-5-methoxybenzoic acid (yellow circles) and gallic acid (grey circles). During growth on syringate, gallic acid is produced via 3,4-dihydroxy-5-methoxybenzoic acid as an intermediate, a pathway similarly observed in other anaerobes[1]. However, due to the instability of gallic acid in solution, accurate concentration measurements could not be obtained. Data are presented as mean +/− standard deviation as error bars (n = 3 biological replicates). Doubling times (t_d) were calculated for each condition. The theoretical ratio of methyl donor to acetate is 4:3 ($4\ CH_3Cl + 2\ CO_2 + 2\ H_2O \rightarrow 3\ CH_3COO^- + 7\ H^+ + 4\ Cl^-$; $\Delta G^{\circ\prime} = -419.9$ kJ mol^{-1} [2]), which is

consistent with the observed ratios for most of the growth curves considering that about 10% of the available carbon is used for anabolism. It is noteworthy that syringate possesses two methyl groups, whereas CM has one. For panel 1C comparatively less acetate was produced, indicating that additional fermentation products might have been produced. Source data are provided as a Source Data file.

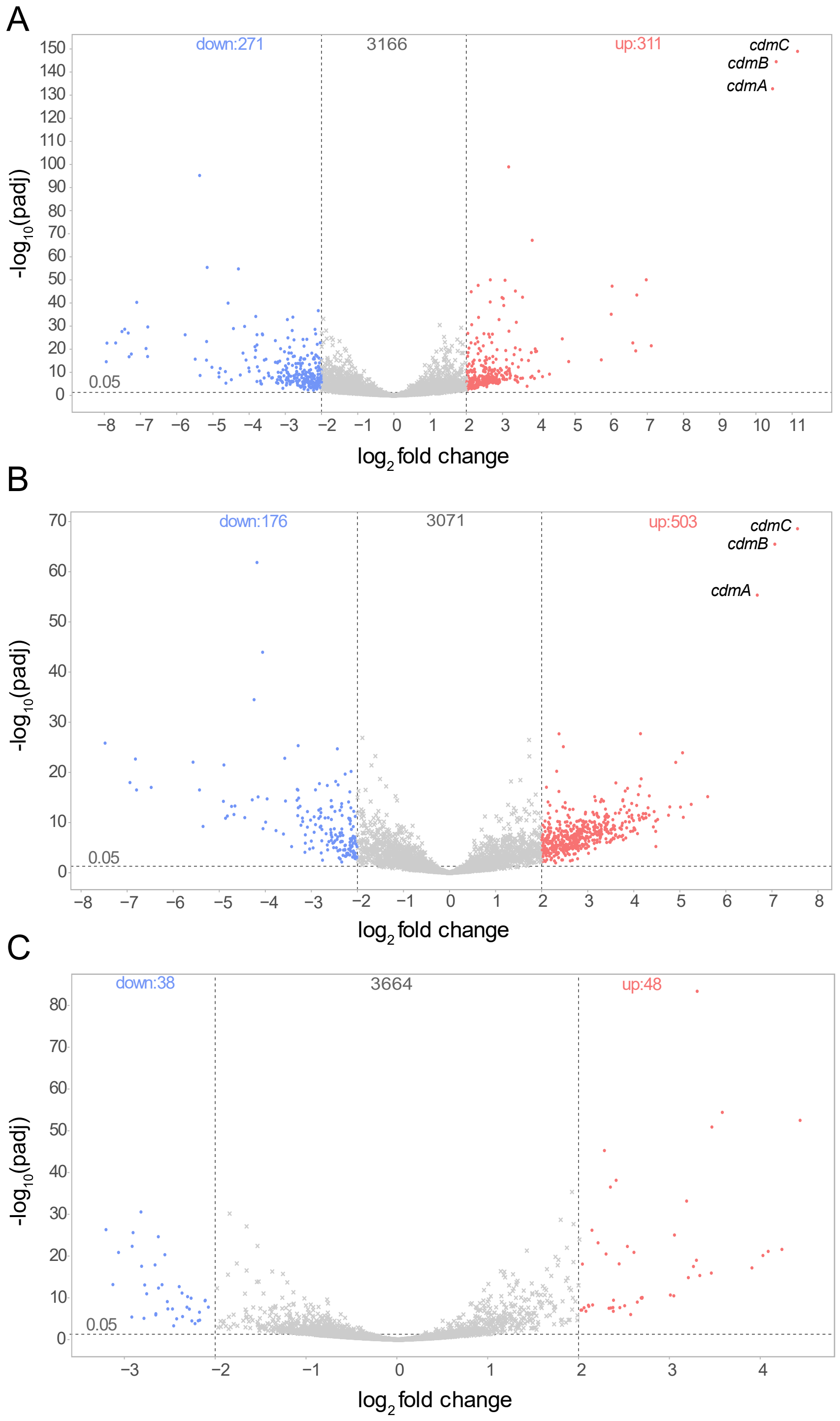

Supplementary Figure 2. Volcano plot showing differential gene expression during growth of *A. dehalogenans* on CM and syringate. The plots compare gene expression profiles between CM and syringate (**A**), syringate + CM versus syringate (**B**) and CM versus syringate + CM (**C**) (n = 3 biological replicates). Gene expression categories are color-coded: grey (non-differentially expressed), blue (significantly downregulated), and red (significantly upregulated). padj: adjusted p-value. P-values were calculated using the two-sided Wald test (DESeq2) and adjusted for multiple testing with the Benjamini-Hochberg method. Source data are provided in Supplementary Data 1.

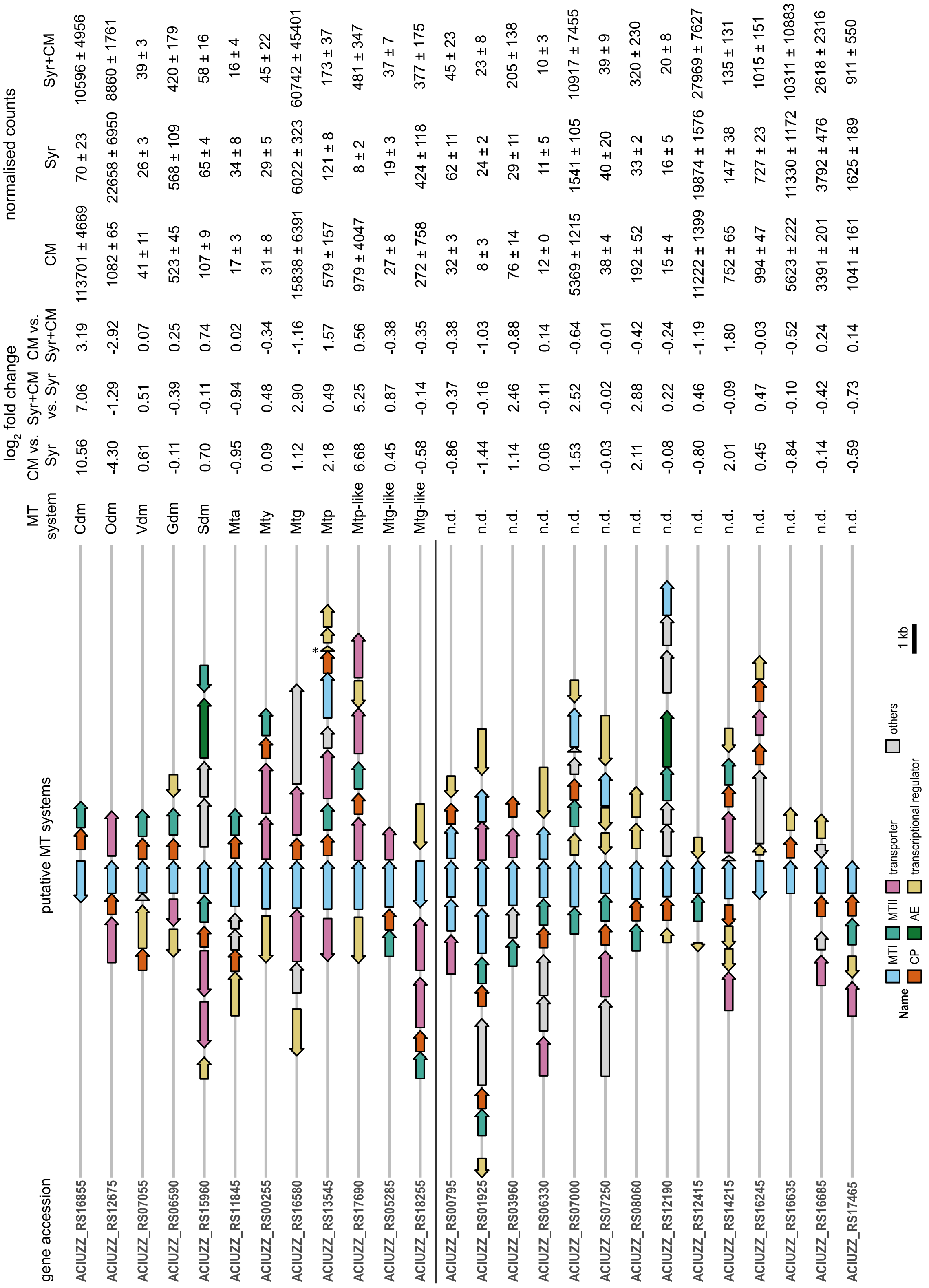

Supplementary Figure 3. Methyltransferase (MT) systems in *A. dehalogenans*, including their genetic organisation and differential expression values of the methyltransferase I (MTI) upon growth with CM and syringate (Syr). The left panel displays the gene accession number of the putative MTIs. The depicted methyltransferase systems comprise *O*-demethylases for vanillate (Odm)[3], veratrole (Vdm)[4], guaiacol (Gdm)[5], and syringate (Sdm)[5], as well as the putative methyltransferases for methanol (Mta)[6,7], γ-butyrobetaine (Mty, 76% sequence identity to *Eubacterium limosum* MtyB (WP_013382626.1)[8]), glycin-betain methyltransferase (Mtg)[9], and proline betaine (Mtp)[10]. Additional methyltransferase systems include Mtp-like methyltransferases (51% sequence identity to *Eubacterium*

limosum MtpB (WP_038353400.1)[10]), Mtg-like methyltransferases (31% sequence identity to *Desulfitobacterium hafniense* Mtg (WP_005816521)[9]), as well as several yet uncharacterised methyltransferase systems with unknown (n.d.) substrates. Genes encoding MTI are shown in light blue, genes for the corrinoid protein (CP) in orange, genes for methyltransferase II (MTII) in turquoise and genes for the corrinoid activating enzyme (AE) in dark green. Transporters and transcription regulators are shown in pink and yellow, respectively, while genes with other annotations appear in grey. Pseudogenes are marked with asterisks. The right panel shows the $\log_2$ fold changes between CM and Syringate (Syr), Syr + CM vs. Syr, and CM vs. Syr + CM, as well as the normalised counts for the aligned MTIs of each MT systems shown (n = 3 biological replicates). P-values were calculated using the two-sided Wald test (DESeq2) and adjusted for multiple testing with the Benjamini-Hochberg method. See Supplementary Data 1 for additional data.

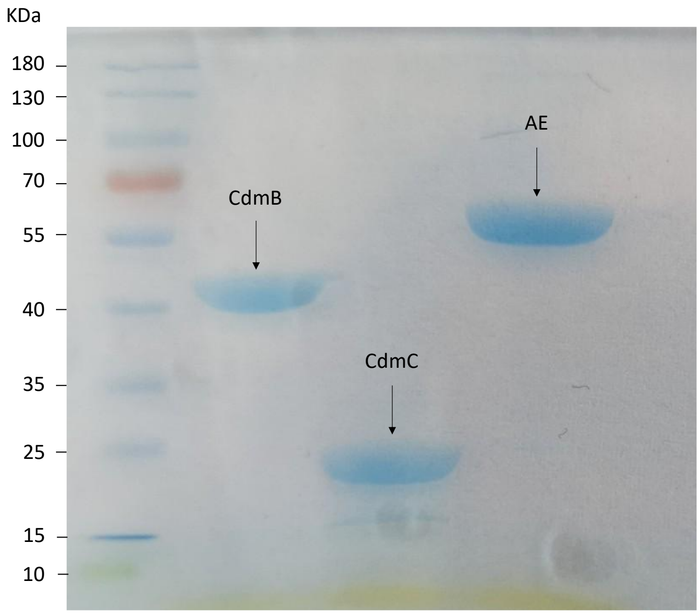

Supplementary Figure 4. SDS-PAGE gel of purified CdmB, CdmC and AE. Protein samples (approximately 1 mg each) were resolved on an 12.5 % SDS gel to assess purity and confirm expected molecular weights: CdmB (~47 kDa), CdmC (~22 kDa), and AE (~65 kDa). Source data are provided as a Source Data file.

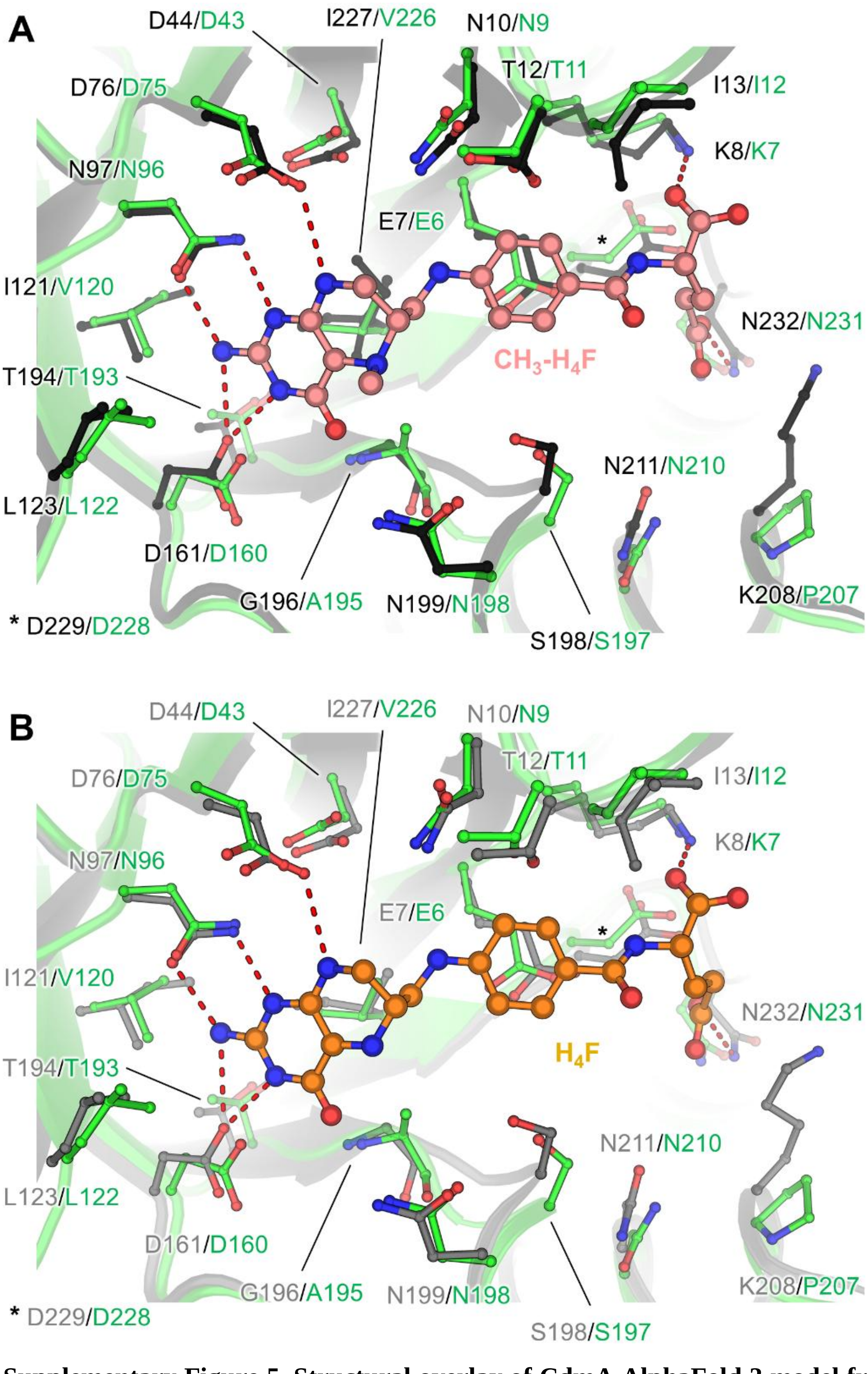

Supplementary Figure 5. Structural overlay of CdmA AlphaFold 3 model from *A. dehalogenans* with the methyltransferase involved in *O*-demethylation from *Desulfitobacterium hafniense* DCB-2[11]. A, Superposition of CdmA (green) with the methyltransferase from *D. hafniense* containing the methyl-tetrahydrofolate (black, PDB: 4O1E [https://doi.org/10.2210/pdb4O1E/pdb]). **B**, Superposition of CdmA (green) with the methyltransferase from *D. hafniense* containing the tetrahydrofolate (grey, PDB: 4O1F [https://doi.org/10.2210/pdb4O1F/pdb]). In both figures, the surrounding residues in the close vicinity of the ligand are shown as sticks with the (methyl-)tetrahydrofolate displayed as balls and sticks. The hydrogen bond network between the methyltransferase of *D. hafniense* and the ligands is shown by red dashes. CH_3-H_4F: methyl-tetrahydrofolate, H_4F: tetrahydrofolate.

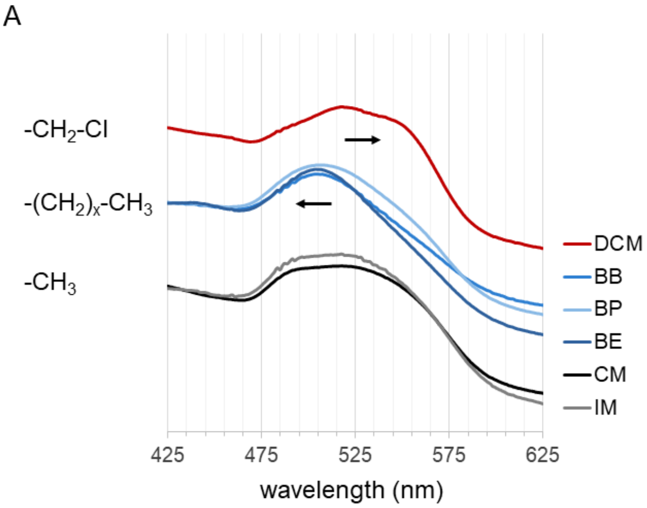

B

	Carbon & energy source		
Haloalkane	-	CM	Syr
DCM	0.061 ± 0.002	0.069 ± 0.006	0.628 ± 0.033
BE	0.023 ± 0.002	0.023 ± 0.005	0.043 ± 0.010
BP	0.018 ± 0.006	0.026 ± 0.003	0.039 ± 0.011
BB	0.034 ± 0.006	0.016 ± 0.004	0.047 ± 0.004

Supplementary Figure 6. UV-vis spectra of Co(III)-CdmC following reaction with various haloalkanes (A) and OD_{max} values at 578 nm for growth of *A. dehalogenans* in the presence of dichloromethane (DCM) and longer-chain haloalkanes (B). A, To assess the activity of CdmB towards different haloalkanes (Table 1), various substrates were added to Co(I)-CdmC. The subsequent formation of Co(III)-CdmC is indicated by a peak at approximately 520 nm. Compared to the methyl halides chloromethane (CM) and iodomethane (IM), the peak is blue-shifted for bromoethane (BE), 1-bromopropane (BP), and 1-bromobutane (BB), and red-shifted for DCM. The peak shift suggests different reaction products, potentially forming CH_3-$(CH_2)_X$-Co(III) or Cl-CH_2-Co(III) instead of CH_3-Co(III). Spectra were normalised to 432 nm. **B**, Growth of *A. dehalogenans* on CM, syringate (Syr), or without supplements was monitored in the presence of DCM, bromoethane, 1-bromopropane and 1-bromobutane, and the results are shown as OD_{max} values at 578 nm (n = 3 biological replicates). Growth without CM and syringate is due to growth on yeast extract. Source data are provided as a Source Data file.

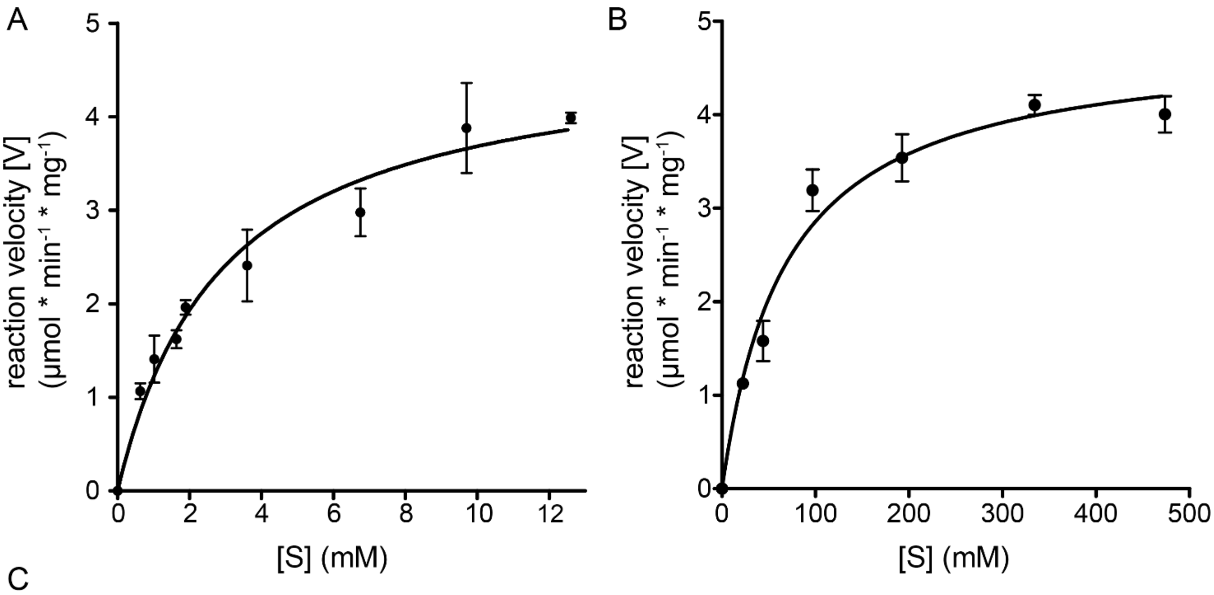

C

Kinetic parameter	CM	DCM
V_{max}	4.76 ± 0.36 µmol·min^{-1}·mg^{-1}	4.81 ± 0.24 µmol·min^{-1}·mg^{-1}
K_m	2.92 ± 0.18 mM	68.67 ± 9.66 mM
k_{cat}	3.82 ± 0.29 s^{-1}	3.86 ± 0.19 s^{-1}
k_{cat}/K_m	1308.16 ± 100.03 M^{-1}·s^{-1}	56.72 ± 5.71 M^{-1}·s^{-1}

Supplementary Figure 7. Michaelis-Menten kinetics for CM (A) and DCM (B). Data are presented as mean +/− standard deviation as error bars (n = 3 technical replicates). Nonlinear regression analysis of GraphPad Prism3 was used for curve fitting and to obtain $V_{\max}$ and K_{m} values **(C)**. Source data are provided as a Source Data file.

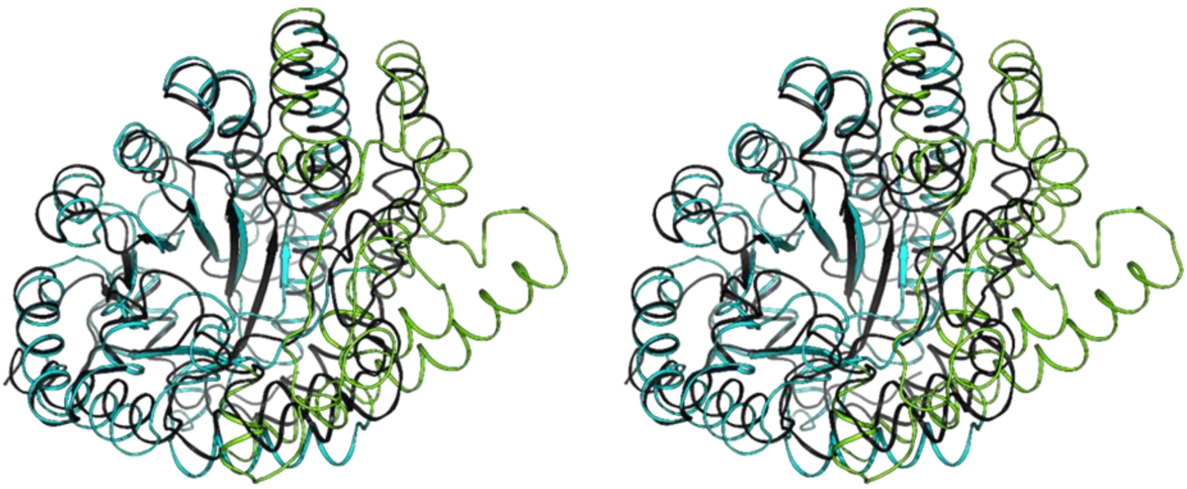

Supplementary Figure 8. Structural alignment between CdmB and the human uroporphyrinogen III decarboxylase UroD. Stereo view of the structural superposition between CdmB apoprotein (cyan and green) and UroD (black, PDB code 1JPH [https://doi.org/10.2210/pdb1JPH/pdb]) represented in cartoon. The cyan colour highlights the TIM barrel core of CdmB, and the green highlights the extensions located between the first three β-sheets. The superposition has been done on the core TIM barrel of CdmB coloured in cyan.

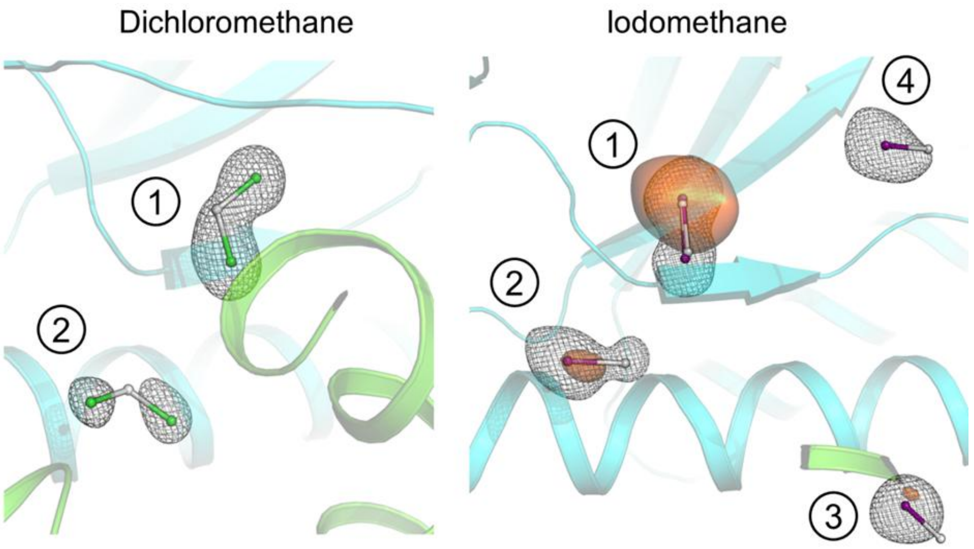

Supplementary Figure 9. Additional electron density observed in the models from the CdmB crystals soaked with haloalkanes. Cartoon representation of CdmB with the core TIM barrel in cyan and specific extensions in green. The ligands (DCM left, and CH_3I right) are displayed as balls and sticks with carbon, chloride, and iodine coloured white, green, and deep purple, respectively. The electron density $2F_o - F_c$ is contoured to 1 σ. For the CH_3I, the anomalous map of the dataset collected at 7.1 keV is displayed as a transparent orange surface and contoured to 4.5 σ. Sites shown in Figure 3 are added. Site 1 harbours a mixture of CH_3I modelled in the opposite orientation, with one preferential orientation.

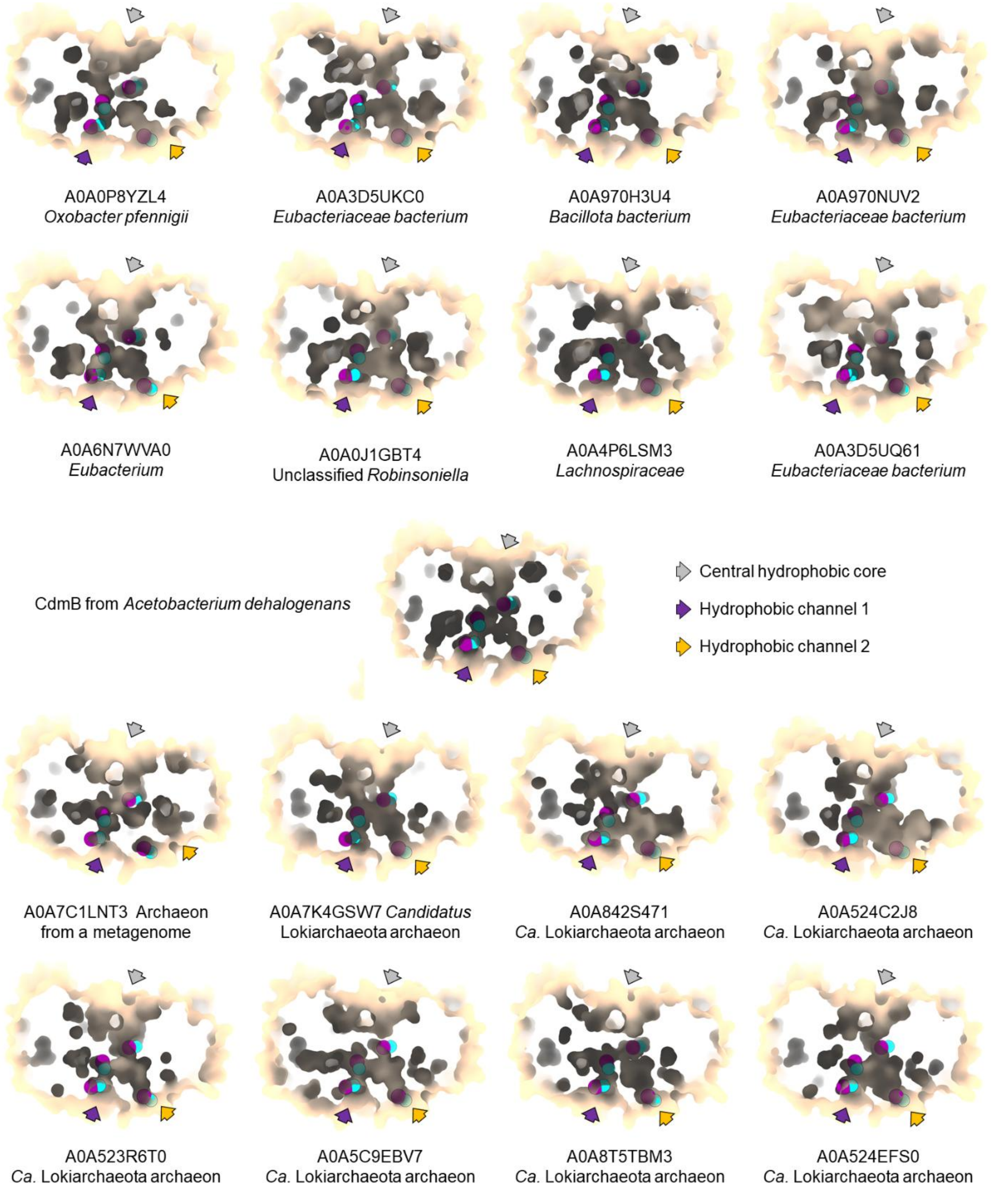

Supplementary Figure 10. AlphaFold 3 models of close CdmB homologues highlighting tunnelling conservation. AlphaFold 3 models were generated for the 16 homologs of CdmB (Supplementary Data 2) and are shown as surfaces, with the exterior coloured yellow and the hollow hydrophobic cavity in grey. Arrows point to the different tunnels and clefts. The CH_3I observed in the experimental CdmB structure is shown as balls.

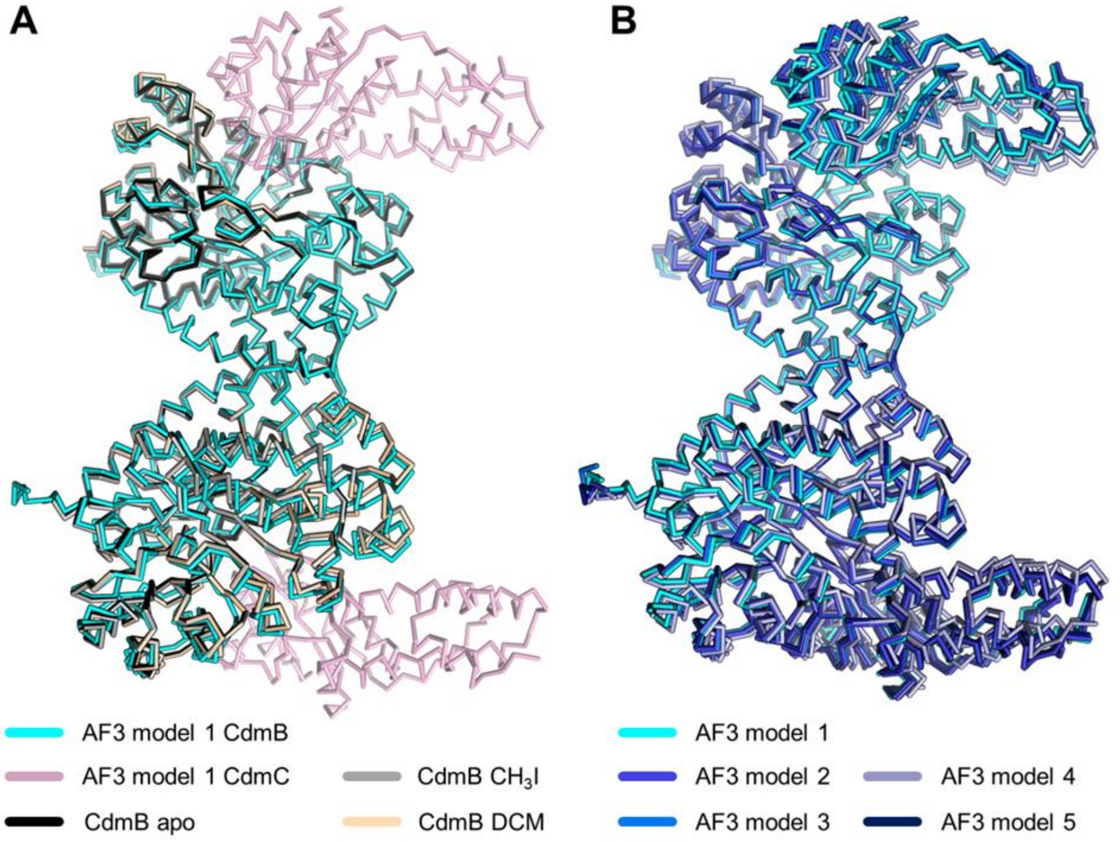

Structural alignment with CdmB apo

Model	rmsd (Å)	rmsd (aligned Cα)
CdmB CH_3I	0.13	829
CdmB DCM	0.11	831
AF3 model 1	0.95	778
AF3 model 2	0.74	744
AF3 model 3	0.73	768
AF3 model 4	0.63	776
AF3 model 5	0.78	794

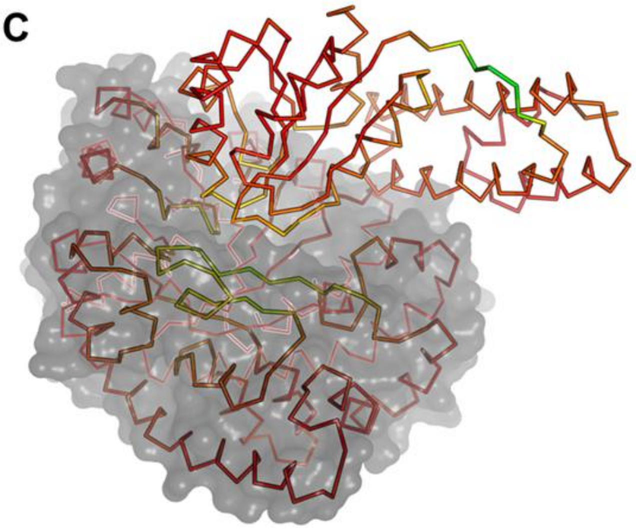

Supplementary Figure 11. Superposition of the AlphaFold 3 CdmBC models and confidence score. A, All structures are represented in ribbon and show the structural superposition of CdmB apo with experimental and AlphaFold 3 (AF3) best model 1. **B,** Superposition of all generated AF3 models. **C,** Confidence score of the AF3 model 1, ranging from red (high confidence) to green (low confidence). The CdmC linker region connecting the N-terminal four helix bundle domain to the Rossmann domain (residues 83-91) and the CdmB β-sheet 116-138 has the lowest confidence score, which hints at their natural flexibility. The β-sheet 116-138 might serve as a lid to allow the Rossmann fold domain to penetrate deeper into the central TIM barrel cavity, thereby isolating the reactive Co(I)-B_{12} from the solvent and placing it in front of site 4 at a close distance to the halogenated substrate before the nucleophilic attack.

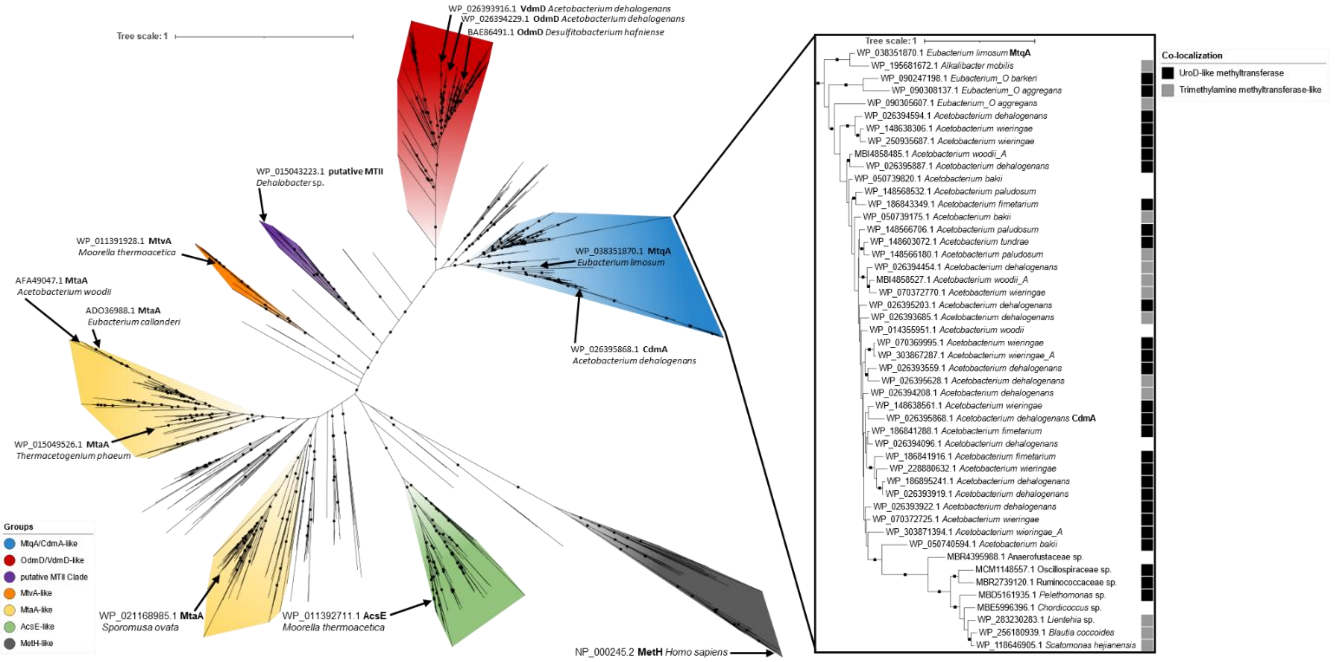

Supplementary Figure 12. Phylogenetic tree displaying the relationship among CdmA and related MTIIs, including OdmD, VdmD, MtvA, MtaA, the subunit AcsE from the carbon monoxide dehydrogenase/acetyl-CoA synthase complex and methionine synthase MetH. MetH is proposed as the outgroup. Branch support is indicated by black squares, with ultrafast bootstraps and Shimodaira-Hasegawa approximate likelihood-ratio test values above 95 and 80, respectively. MTIIs which are syntenic with UroD-like and trimethylamine MTIs genes (up to five genes up-/downstream) are highlighted with black (UroD-like methyltransferase) and grey (trimethylamine methyltransferase) squares, respectively. The putative MTII clade comprises MTIIs which are co-localized with uncharacterised CdmB homologues from haloalkane-degrading bacteria. Source data are provided in Supplementary Data 3.

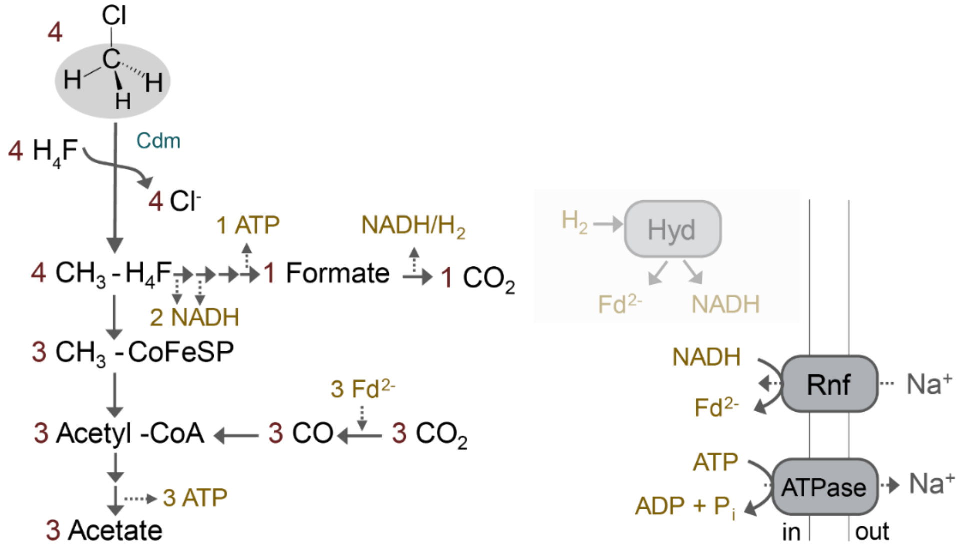

Supplementary Figure 13. A tentative pathway for CH_3-H_4F conversion to acetate is proposed, involving electron transfer coupled to membrane potential generation similar to that described for *A. woodii*[12,13]: Rnf complex (ACIUZZ_RS11550-75, ACIUZZ_RS17540-65), electron bifurcating hydrogenase HydABC (ACIUZZ_RS04275-95), ATPase (F_1F_0 ATPase ACIUZZ_RS01065-115, V-type ATPase ACIUZZ_RS06220-55), WLP enzymes (ACIUZZ_RS15910-35), CODH/ACS (ACIUZZ_RS15200-45), formate dehydrogenase (ACIUZZ_RS00730, ACIUZZ_RS03155).

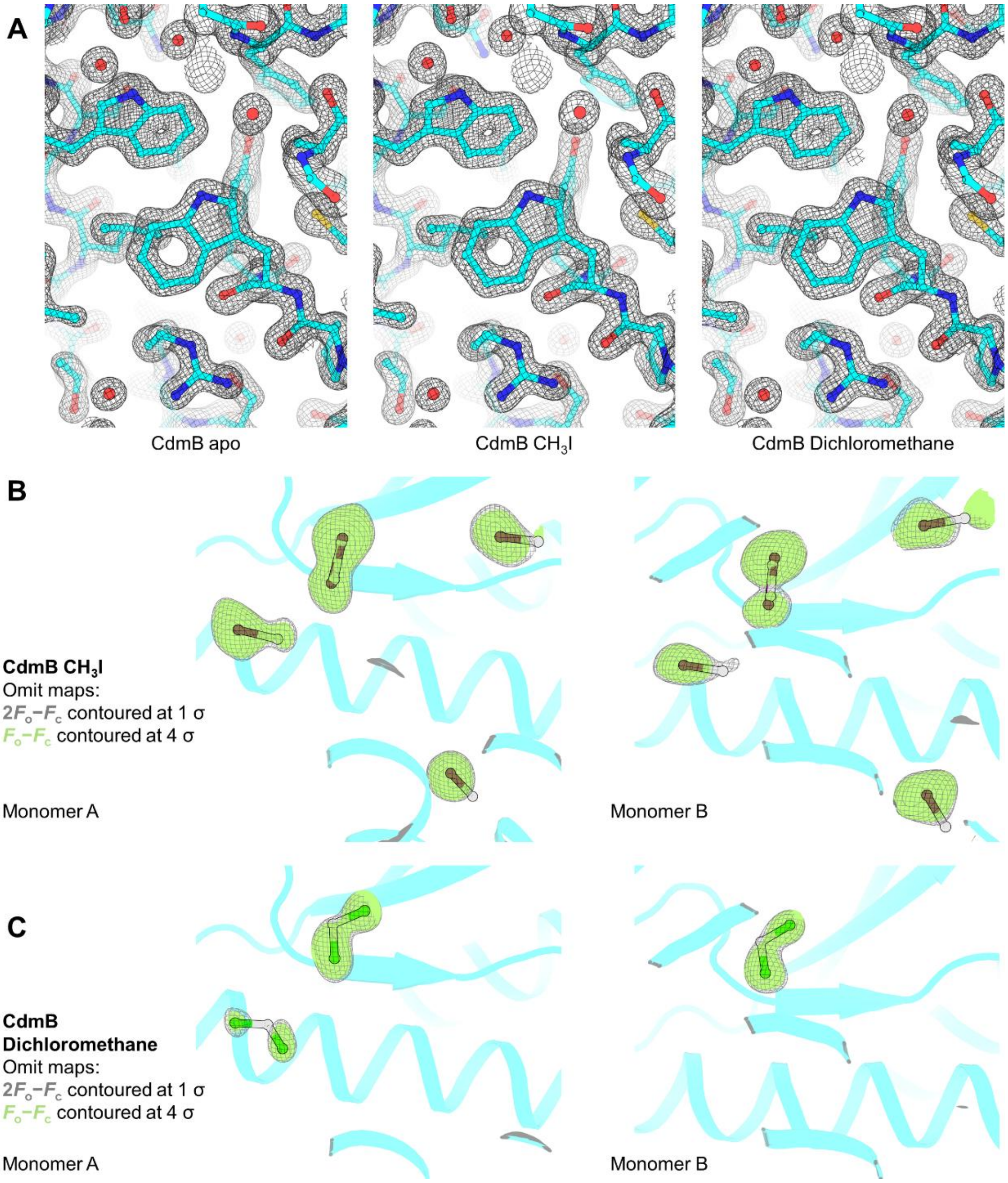

Supplementary Figure 14. Electron density maps of CdmB and omit maps of the ligands. **A**, Electron density quality for the different CdmB models. CdmB is shown as sticks, with water molecules as red spheres. The $2F_o{-}F_c$ map shown as a grey mesh is contoured at 2-σ. B and C, omit maps for the CH_3I (**B**) and dichloromethane (**C**) in which the 2 $F_o{-}F_c$ maps (grey mesh) resulting from the omission of the ligands are contoured at 1-σ and the positive $F_o{-}F_c$ maps (chartreuse transparent surface) are contoured at 4-σ. The protein backbone is shown as a transparent cyan cartoon, and the ligands are shown in balls and sticks. Atoms are coloured as follows: carbon in cyan/white, nitrogen in blue, oxygen in red, sulphur in yellow, chloride in green, and iodine in purple.

Supplementary Table 1: X-ray analysis statistics.

	CdmB apoprotein	CdmB with iodomethane	CdmB with DCM	CdmB apoprotein 7.1 keV	CdmB with iodomethane 7.1 keV
**Data collection**					
Wavelength (Å)	0.97951	0.97951	0.97951	1.74626	1.74626
Space group	*C*2	*C*2	*C*2	*C*2	*C*2
Resolution (Å)	153.86 – 1.57 (1.72 – 1.57)	77.02 – 1.62 (1.79 – 1.62)	154.22 – 1.62 (1.75 – 1.62)	52.67 – 2.31 (2.35 – 2.31)	53.61 – 2.32 (2.36 – 2.32)
Cell dimensions					
a, b, c (Å)	69.51, 85.79, 155.80	69.52, 86.15, 156.07	69.57, 86.06, 156.27	69.50, 85.87, 155.97	69.50, 85.99, 155.92
α, β, γ (°)	90, 99.05, 90	90, 99.27, 90	90, 99.29, 90	90, 99.07, 90	90, 99.34, 90
R_{merge} (%)[a]	6.2 (123.0)	7.5 (141.0)	6.4 (125.6)	12.7 (168.9)	14.3 (208.3)
R_{pim} (%)[a]	2.5 (49.8)	2.8 (51.4)	2.9 (52.9)	8.0 (111.0)	9.2 (136.3)
$CC_{1/2}$ [a]	0.999 (0.530)	0.999 (0.456)	0.999 (0.401)	0.996 (0.556)	0.996 (0.419)
I/σ_I[a]	15.7 (1.5)	13.0 (1.4)	13.6 (1.5)	5.8 (1.1)	5.3 (0.8)
Spherical completeness[a]	65.4 (13.5)	66.7 (12.7)	76.8 (19.3)	96.8 (94.1)	95.6 (98.2)
Ellipsoidal completeness[a]	90.6 (57.2)	91.6 (59.8)	92.8 (56.5)	/	/
Redundancy[a]	7.0 (6.8)	8.0 (8.4)	5.9 (6.5)	6.7 (6.0)	6.4 (6.3)
Nr. unique reflections[a]	82,269 (4,113)	76,703 (3,835)	87,876 (4,394)	38,713 (1,827)	37,637 (1,858)
**Refinement**					
Resolution (Å)	27.88 – 1.57	30.92 – 1.62	34.33 – 1.62		
Number of reflections	82,182	76,597	87,810		
R_{work}/R_{free}[b] (%)	17.33/19.53	17.78/19.89	16.23/19.06		
Number of atoms					
Protein	6,699	6,739	6,708		
Ligands/ions	246	299	344		
Solvent	502	469	444		
Mean B-value ($Å^2$)	37.47	37.42	38.49		
Molprobity clash score	1.58	1.27	0.70		
Ramachandran plot					
Favoured regions (%)	97.36	97.72	97.72		
Outlier regions (%)	0	0	0		
rmsd[c] bond lengths (Å)	0.008	0.007	0.011		
rmsd[c] bond angles (°)	1.000	0.908	1.140		
**PDB code**	9RUI	9RUO	9RUL		

[a] Values relative to the highest resolution shell are within parentheses. [b] Rfree was calculated as the Rwork for 5% of the reflections that were not included in the refinement. [c] rmsd, root mean square deviation.

Supplementary References

1. Wu, Z.R., Daniel, S.L. & Drake, H.L. Characterization of a CO-dependent *O*-demethylating enzyme system from the acetogen *Clostridium thermoaceticum*. *Journal of Bacteriology* **170**, 5747-5750 (1988).
2. Traunecker, J., Preu, A. & Diekert, G. Isolation and characterization of a methyl chloride utilizing, strictly anaerobic bacterium. *Archives of Microbiology* **156**, 416–421 (1991).
3. Kaufmann, F., Wohlfarth, G. & Diekert, G. *O*-demethylase from *Acetobacterium dehalogenans* - cloning, sequencing, and active expression of the gene encoding the corrinoid protein. *European Journal of Biochemistry* **257**, 515-521 (1998).
4. Engelmann, T., Kaufmann, F. & Diekert, G. Isolation and characterization of a veratrol:corrinoid protein methyl transferase from *Acetobacterium dehalogenans*. *Archives of Microbiology* **175**, 376–383 (2001).
5. Nguyen, H.D., Studenik, S. & Diekert, G. Corrinoid activation by a RACE protein: studies on the interaction of the proteins involved. *FEMS Microbiology Letters* **345**, 31-38 (2013).
6. Sauer, K. & Thauer, R.K. Methanol:coenzyme M methyltransferase from *Methanosarcina barkeri*. Zinc dependence and thermodynamics of the methanol:cob(I)alamin methyltransferase reaction. *European Journal of Biochemistry* **249**, 280-285 (1997).
7. Kremp, F. & Müller, V. Methanol and methyl group conversion in acetogenic bacteria: biochemistry, physiology and application. *FEMS Microbiology Reviews* **45**, fuaa040 (2021).
8. Ellenbogen, J.B., Jiang, R., Kountz, D.J., Zhang, L. & Krzycki, J.A. The MttB superfamily member MtyB from the human gut symbiont *Eubacterium limosum* is a cobalamin-dependent γ-butyrobetaine methyltransferase. *The Journal of Biological Chemistry* **297**, 101327 (2021).
9. Ticak, T., Kountz, D.J., Girosky, K.E., Krzycki, J.A. & Ferguson, D.J. A nonpyrrolysine member of the widely distributed trimethylamine methyltransferase family is a glycine betaine methyltransferase. *Proceedings of the National Academy of Sciences USA* **111**, E4668-E4676 (2014).
10. Picking, J.W., Behrman, E.J., Zhang, L. & Krzycki, J.A. MtpB, a member of the MttB superfamily from the human intestinal acetogen *Eubacterium limosum*, catalyzes proline betaine demethylation. *The Journal of Biological Chemistry* **294**, 13697–13707 (2019).
11. Sjuts, H., Dunstan, M.S., Fisher, K. & Leys, D. Structures of the methyltransferase component of *Desulfitobacterium hafniense* DCB-2 *O*-demethylase shed light on methyltetrahydrofolate formation. *Acta Crystallographica Section D* **71**, 1900-1908 (2015).
12. Westphal, L., Wiechmann, A., Baker, J., Minton, N.P. & Müller, V. The Rnf complex is an energy-coupled transhydrogenase essential to reversibly link cellular NADH and ferredoxin pools in the acetogen *Acetobacterium woodii*. *Journal of Bacteriology* **200**, e00357-18 (2018).
13. Schuchmann, K. & Müller, V. Autotrophy at the thermodynamic limit of life: a model for energy conservation in acetogenic bacteria. *Nature Reviews Microbiology* **12**, 809-821 (2014).
