# Supplementary material for: Identification and characterisation of an elusive bacterial enzyme system for chloromethane dehalogenation
Source: Nat Commun. 2026 May 30;17:4818. doi: 10.1038/s41467-026-73764-z (PMC13222342; doi:10.1038/s41467-026-73764-z)
Supplement: Supplementary file 2 — Description of Additional Supplementary File [file 41467_2026_73764_MOESM2_ESM.pdf]

The Description of Additional Supplementary Files

Supplementary Data 1: Raw count data, log2 fold change values and adjusted p-values generated during transcriptomic analysis within this study as well as transcriptomic data specifically used for generating the heatmap (Fig. 1) and the overview of A. dehalogenans methyltransferase systems (Supplementary Figure 3)

Supplementary Data 2: CdmB homologues used to compute the conservation score for CdmB displayed in Fig. 3 and to generate the AlphaFold 3 models shown in Supplementary Fig. 10.

Supplementary Data 3: BLASTp queries and data used for Cdm system completeness prediction for Fig. 5 and Supplementary Fig. 12.
