# Supplementary material for: Identification and characterisation of an elusive bacterial enzyme system for chloromethane dehalogenation
Source: Nat Commun. 2026 May 30;17:4818. doi: 10.1038/s41467-026-73764-z (PMC13222342; doi:10.1038/s41467-026-73764-z)
Supplement: Supplementary file 6 — Reporting Summary [file 41467_2026_73764_MOESM6_ESM.pdf]

Corresponding author(s): Julia Kurth
Last updated by author(s): Apr 24, 2026

Reporting Summary

Nature Portfolio wishes to improve the reproducibility of the work that we publish. This form provides structure for consistency and transparency in reporting. For further information on Nature Portfolio policies, see our Editorial Policies and the Editorial Policy Checklist.

Statistics

For all statistical analyses, confirm that the following items are present in the figure legend, table legend, main text, or Methods section.

n/a	Confirmed	
☐	☒	The exact sample size (n) for each experimental group/condition, given as a discrete number and unit of measurement
☐	☒	A statement on whether measurements were taken from distinct samples or whether the same sample was measured repeatedly
☐	☒	The statistical test(s) used AND whether they are one- or two-sided<br>*Only common tests should be described solely by name; describe more complex techniques in the Methods section.*
☒	☐	A description of all covariates tested
☒	☐	A description of any assumptions or corrections, such as tests of normality and adjustment for multiple comparisons
☐	☒	A full description of the statistical parameters including central tendency (e.g. means) or other basic estimates (e.g. regression coefficient) AND variation (e.g. standard deviation) or associated estimates of uncertainty (e.g. confidence intervals)
☐	☒	For null hypothesis testing, the test statistic (e.g. F, t, r) with confidence intervals, effect sizes, degrees of freedom and P value noted<br>*Give P values as exact values whenever suitable.*
☒	☐	For Bayesian analysis, information on the choice of priors and Markov chain Monte Carlo settings
☒	☐	For hierarchical and complex designs, identification of the appropriate level for tests and full reporting of outcomes
☒	☐	Estimates of effect sizes (e.g. Cohen's d, Pearson's r), indicating how they were calculated

Our web collection on statistics for biologists contains articles on many of the points above.

Software and code

Policy information about availability of computer code

Data collection

Liquid chromatography: MassHunter version 10.0
Gas chromatography: LabSolution version 5.124 SP1 and PeakSimple version 4.54
Chloride assay: Gen5 version 3.12.02
UV-vis-spectroscopy and time course measurements: Cary WinUV version 5.3

Data analysis

Growth and metabolite analysis:
Microsoft Office Excel Professional Plus 2016

RNA sequencing and data analysis:
NovaSeq Control Software v1.7
trim_galore v0.6.7, Cutadapt v4.1, FastQC v0.11.9, Bowtie2 v2.5.0, featureCounts (subread v2.0.3), DESeq2 v1.38.0

Enzyme activity/kinetics:
GraphPad Prism 3

Phylogenetic analysis:
CD-Hit v4.8.1, Clustal Omega v1.2.4, AliView v2021, IQ-TREE 2 v2.4.0, ModelFinder, iTOL v7

Structure determination, refinement, and model validation:
autoPROC 1.0.5, PHENIX v. 1.19.2-4158, COOT version 0.9.8.3, PyMOL Version 2.2.0, AlphaFold 3,

For manuscripts utilizing custom algorithms or software that are central to the research but not yet described in published literature, software must be made available to editors and reviewers. We strongly encourage code deposition in a community repository (e.g. GitHub). See the Nature Portfolio guidelines for submitting code & software for further information.

Data

Policy information about availability of data

All manuscripts must include a data availability statement. This statement should provide the following information, where applicable:

- Accession codes, unique identifiers, or web links for publicly available datasets
- A description of any restrictions on data availability
- For clinical datasets or third party data, please ensure that the statement adheres to our policy

Transcriptomics data were deposited under GenBank SRR32341242-SRR32341250. The raw sequencing files from this study are available at the NCBI Sequence Read Archive (SRA) under BioProject ID PRJNA1223667, including BioSample accessions SRR32341242-SRR32341250 (https://www.ncbi.nlm.nih.gov/bioproject/PRJNA1223667). Plots and normalised count tables generated from this analysis are available on Zenodo (DOI: 10.5281/zenodo.19634717). Phylogenetic trees and trimmed alignments are available on figshare (https://doi.org/10.6084/m9.figshare.29852144). The CdmB models and their associated structure factors were deposited in the Protein Data Bank under the following accession codes: 9RUI [https://doi.org/10.2210/pdb9RUI/pdb] (CdmB apo); 9RUL [https://doi.org/10.2210/pdb9RUL/pdb] (CdmB soaked with DCM); and 9RUO [https://doi.org/10.2210/pdb9RUO/pdb] (CdmB soaked with iodomethane). In the manuscript we also refer to PDB codes 3BUL [https://doi.org/10.2210/pdb3BUL/pdb], 4O1E [https://doi.org/10.2210/pdb4O1E/pdb] and 4O1F [https://doi.org/10.2210/pdb4O1F/pdb]. The HPLC-MS/MS raw data have been deposited on Figshare (https://doi.org/10.6084/m9.figshare.32089645). The raw data from UV/Vis spectroscopy, OD578 measurements, chloride assay, GC and activity assay measurements as well as the processed HPLC-MS data generated in this study are provided in the Source Data file. Source Data are provided with this paper.

Research involving human participants, their data, or biological material

Policy information about studies with human participants or human data. See also policy information about sex, gender (identity/presentation), and sexual orientation and race, ethnicity and racism.

Reporting on sex and gender	no human participants were used in this study
Reporting on race, ethnicity, or other socially relevant groupings	no human participants were used in this study
Population characteristics	no human participants were used in this study
Recruitment	no human participants were used in this study
Ethics oversight	no human participants were used in this study

Note that full information on the approval of the study protocol must also be provided in the manuscript.

Field-specific reporting

Please select the one below that is the best fit for your research. If you are not sure, read the appropriate sections before making your selection.

☒ Life sciences ☐ Behavioural & social sciences ☐ Ecological, evolutionary & environmental sciences

For a reference copy of the document with all sections, see nature.com/documents/nr-reporting-summary-flat.pdf

Life sciences study design

All studies must disclose on these points even when the disclosure is negative.

Sample size	No formal sample size calculation was performed. Sample sizes were determined based on experimental feasibility and established practice for microbial growth experiments, transcriptomic data analysis, and enzymatic activity determination. For growth experiments, including GC, LC and chloride assay measurements, as well as for transcriptomic analysis, biological triplicates were chosen. Enzyme activity assays for activity calculations were conducted in triplicates.
Data exclusions	No data was excluded
Replication	All replication attempts were successfull.
Randomization	Randomization was applied to generate the Rfree set for the structural refinement. Except for structural analyses, randomization was not applied.
Blinding	Blinding was not relevant to this study.

Reporting for specific materials, systems and methods

We require information from authors about some types of materials, experimental systems and methods used in many studies. Here, indicate whether each material, system or method listed is relevant to your study. If you are not sure if a list item applies to your research, read the appropriate section before selecting a response.

Materials & experimental systems

n/a	Involved in the study
☒	☐ Antibodies
☒	☐ Eukaryotic cell lines
☒	☐ Palaeontology and archaeology
☒	☐ Animals and other organisms
☒	☐ Clinical data
☒	☐ Dual use research of concern
☒	☐ Plants

Methods

n/a	Involved in the study
☒	☐ ChIP-seq
☒	☐ Flow cytometry
☒	☐ MRI-based neuroimaging

Plants

Seed stocks	no seed stocks were used
Novel plant genotypes	no plants were used
Authentication	no plants or seed stocks were used
